# SUSD2 Proteolytic Cleavage Requires the GDPH Sequence and Inter-Fragment Disulfide Bonds for Surface Presentation of Galectin-1 on Breast Cancer Cells

**DOI:** 10.3390/ijms20153814

**Published:** 2019-08-05

**Authors:** Mitch E. Patrick, Kristi A. Egland

**Affiliations:** Cancer Biology & Immunotherapies Group, Sanford Research, Sanford School of Medicine of the University of South Dakota, Sioux Falls, SD 57104, USA

**Keywords:** breast cancer, SUSD2, galectin-1

## Abstract

Galectin-1 (Gal-1) is a 14 kDa protein that has been well characterized for promoting cancer metastasis and tumor immune evasion. By localizing to the cancer cell surface, Gal-1 induces T cell apoptosis through binding T cell surface receptors. The transmembrane protein, Sushi Domain Containing 2 (SUSD2), has been previously shown to be required for Gal-1 surface presentation in breast cancer cells. Western immunoblot analysis revealed that SUSD2 is cleaved into two fragments. However, the significance of this cleavage for Gal-1 surface localization has not been investigated. To define the location of cleavage, a mutagenesis analysis of SUSD2 was performed. Our studies demonstrated that SUSD2 is cleaved at its glycine-aspartic acid-proline-histidine (GDPH) amino acid sequence. Generation of a noncleavable SUSD2 mutant (GDPH∆-SUSD2) showed that SUSD2 cleavage was required for SUSD2 and Gal-1 plasma membrane localization. Noncleavable cysteine mutants were also unable to present Gal-1 at the cell surface, further demonstrating that SUSD2 cleavage is required for Gal-1 surface presentation. Treatment with the serine protease inhibitor, Pefabloc SC, inhibited SUSD2 cleavage in a dose dependent manner, suggesting that SUSD2 is cleaved by a serine protease. Therefore, identification and inhibition of this protease may provide a new therapeutic tool for inhibiting SUSD2 and Gal-1′s combined tumorigenic function in breast cancer.

## 1. Introduction

Sushi Domain Containing 2 (SUSD2) has been studied in cancer, neurodevelopment, and as a marker for mesenchymal stem cells. In cancer, SUSD2 has been described as both protumor [1,2] and antitumor [3,4] depending on the type of cancer. The mouse homolog of SUSD2, Susd2/SVS-1, has been implicated in the regulation of neurite outgrowth during development [5]. SUSD2 has also been identified as the antigen for the W5C5 antibody, which is used as a marker for the isolation of mesenchymal stem cells from human tonsil and bone marrow [6,7]. While SUSD2 has been studied in diverse biological systems, there are currently few studies on the biochemical properties of the protein. Knowledge of the structure and post-translational processing of SUSD2 may prove useful in determining the functions of the protein in association with health and disease.

Our studies have been focused on the role of SUSD2 in breast cancer. Previous work has shown that 80% of patient breast tumors have moderate to high staining for SUSD2, and healthy tissues have minimal SUSD2 staining [1,8]. In a syngeneic mouse model, tumors with Susd2 had increased angiogenesis and decreased T cells in the tumor microenvironment [1]. These results suggested that SUSD2 is a promising candidate for targeted therapy in breast cancer. SUSD2′s pathogenicity in breast cancer may be mediated through Galectin-1 (Gal-1). SUSD2 interacts with Gal-1 and is necessary for Gal-1 surface presentation [1]. Gal-1 contributes to tumor immune evasion by inducing apoptosis of activated T cells [9]. Kovacs-Solyom et al. demonstrated that Gal-1 surface presentation is required to induce T cell apoptosis [10]. Therefore, SUSD2-mediated surface presentation of Gal-1 may be contributing to tumor immune evasion. To disrupt this process as a potential treatment for breast cancer, a more detailed understanding of cell surface presentation of Gal-1 by SUSD2 is required.

**Figure 1:**
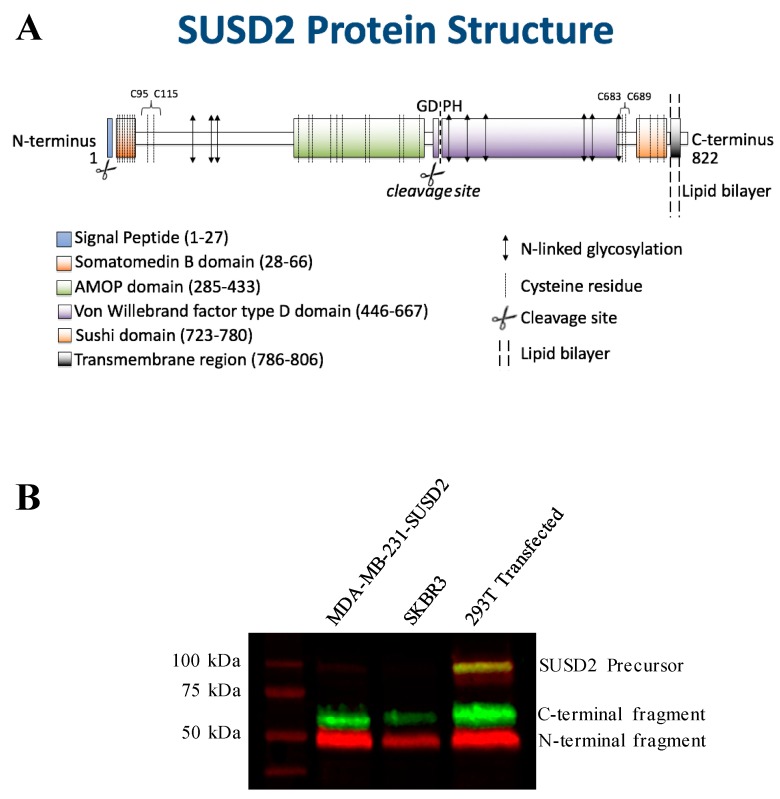
(**A**) SUSD2 is a type I transmembrane protein composed of 822 amino acids (a.a.). The domains are indicated by the colored rectangles as labeled. The a.a. span of each domain is indicated in parentheses. The GDPH sequence is located in the von Willebrand factor type D domain, and is the site of protein cleavage. The double-headed arrows represent N-linked glycosylation sites. Cysteine residues are represented by dashed lines. Specific cysteines are indicated by C followed by the a.a. location. A scissors indicates the site of protein cleavage, and the membrane bilayer is a double-dashed line. (**B**) Western immunoblot analysis of SUSD2. Whole cell lysates were generated from stable MDA-MB-231-SUSD2, wild-type SKBR3, and 293T cells transiently transfected with a SUSD2 expression plasmid. Cell lysates were separated by gel electrophoresis and transferred to a polyvinylidene difluoride (PVDF) membrane. Membranes were probed with anti-N-terminal (red) and anti-C-terminal (green) SUSD2 antibodies. Yellow staining indicates the presence of both SUSD2 fragments.

SUSD2 is an 822-amino acid type 1 transmembrane protein, and it is composed of somatomedin b, adhesion associated domain in MUC4 and other proteins (AMOP), von Willebrand factor type D (VWFD) and sushi domains (Figure 1A). These domains have been implicated in cell adhesion in other proteins, and consistently, SUSD2 has been shown to play a role in cell adhesion functions [5,11]. SUSD2 shares the AMOP and VWFD domains with Mucin 4 (MUC4). A phylogenetic tree generated using AMOP and VWFD domain sequences suggests that SUSD2 and MUC4 developed from a common ancestral gene [12]. The VWFD domain contains a glycine-aspartic acid-proline-histidine (GDPH) amino acid sequence that is conserved in many MUC family members and results in protein cleavage between the aspartic acid and proline residues of the GDPH sequence [13,14,15]. Being a paralog to MUC4, we hypothesized that SUSD2 may have similar processing.

The predicted molecular weight of SUSD2 is 90.4 kDa. However, SUSD2 has nine predicted glycosylation sites (Figure 1A), which would increase the size of the protein. We previously demonstrated by western immunoblot analysis using an anti-SUSD2 antibody that two bands were detected. The larger 110-kDa band was most likely a glycosylated form of SUSD2, and a 60-kDa band suggested that SUSD2 was post-translationally cleaved into two fragments [1]. Since the anti-SUSD2 antibody used as a probe only recognized the C-terminal domain of the protein, only one of the two cleaved fragments was observed. Details of post-translational processing of SUSD2 may reveal critical steps that could be targeted therapeutically to inhibit the function of SUSD2 in breast cancer. Therefore, we investigated the mechanism by which SUSD2 was cleaved and whether the fragments remained associated. Here we report the identification of key post-translational processing steps for SUSD2 that are critical for surface localization of SUSD2 and consequently, Gal-1.

## 2. Results

### 2.1. SUSD2 is Cleaved at the GDPH Sequence

*SUSD2* encodes a type I transmembrane protein that is post-translationally cleaved into two fragments. The site of this cleavage and relevance to the presentation of Gal-1 onto the cell surface has not been investigated. The Egland laboratory has generated several in vitro cell line systems to study the function of SUSD2 [1]. The SKBR3 breast cancer cell line expresses high levels of *SUSD2*. However, MDA-MB-231 cells do not endogenously express *SUSD2* [1]. We previously generated and characterized stable MDA-MB-231-SUSD2 and MDA-MB-231-vector control cell lines [1]. The 293T cell line was also used in this study as a model system to investigate SUSD2 processing. 293T cells do not endogenously express *SUSD2*, therefore, transient transfections of SUSD2-encoding plasmids were performed (Figure 1B). Western immunoblot analysis using anti-N-terminal (red) and anti-C-terminal (green) SUSD2 antibodies revealed that three specific SUSD2 bands were present: 110, 60, and 50 kDa (Figure 1B). This result indicated that full-length SUSD2 (110 kDa, yellow) is cleaved into two fragments (60 kDa, green and 50 kDa, red; Figure 1B).

**Figure 2:**
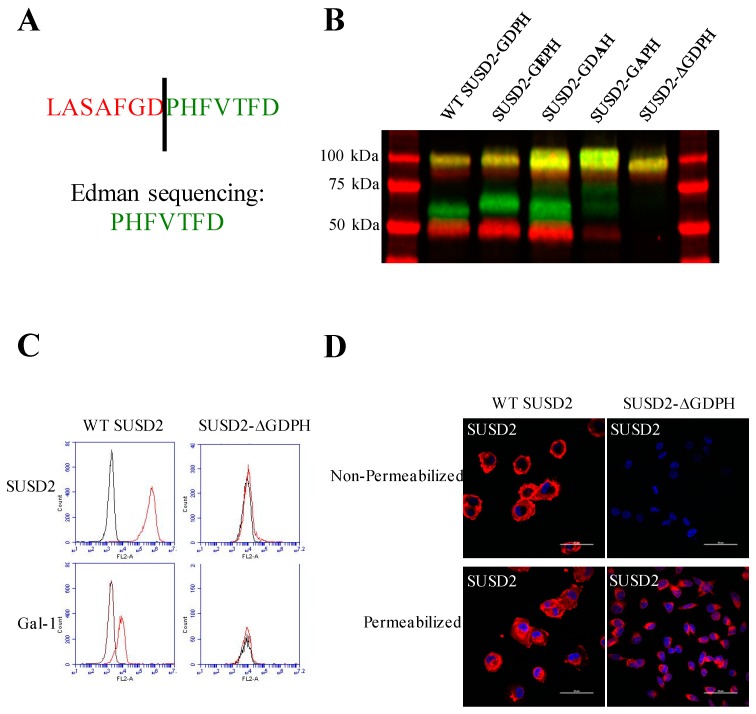
(**A**) SUSD2 sequence at hypothesized cleavage site (top). Edman sequencing results of C-terminal SUSD2 fragment (bottom). (**B**) Western immunoblot analysis of cell lysates generated from transiently transfected 293T cells with expression plasmids encoding WT-SUSD2, SUSD2-GEPH, SUSD2-GDAH, SUSD2-GAPH, or SUSD2-∆GDPH. Anti-C-terminal (green) and anti-N-terminal (red) SUSD2 antibodies were used. (**C**) Effect of SUSD2-∆GDPH deletion on SUSD2 and Gal-1 cell surface localization. Flow cytometry analysis of stable MDA-MB-231-SUSD2 (left) and stable MDA-MB-231-SUSD2-∆GDPH (right) was performed using anti-N-terminal SUSD2 antibody. Cells were not permeabilized, therefore, positive staining indicated presence of cell surface protein. (**D**) SUSD2-∆GDPH remained intercellular and was not localized at the cell surface. Immunofluorescence microscopy of MDA-MB-231-SUSD2 (left) and MDA-MB-231-SUSD2-∆GDPH (right) was performed in nonpermeabilized (top) and permeabilized (bottom) conditions. Cells were stained using anti-SUSD2 antibody and Alexa Fluor 594 secondary antibody (red). Nuclei are stained blue using DAPI.

SUSD2 contains a GDPH sequence within its VWFD domain (Figure 1A). This sequence is conserved in the VWFD domain of MUC4 and other mucin proteins that are cleaved at the GDPH site [13,14,15]. To confirm cleavage of SUSD2 at the GDPH site, we isolated the C-terminal fragment of SUSD2 by polyacrylamide gel electrophoresis and subjected the band to Edman sequencing analysis. Edman sequencing confirmed that SUSD2 was cleaved between the aspartic acid and proline of the GDPH sequence (Figure 2A). SUSD2 constructs encoding mutations within the GDPH sequence were generated to define the required amino acids for cleavage (Figure S1). Since Edman sequencing demonstrated that SUSD2 was cleaved between the aspartic acid and proline of the GDPH sequence, three SUSD2 constructs encoding a single amino acid change in the GDPH sequence were generated, including glycine-glutamic acid-proline-histidine (GEPH), glycine-alanine-proline-histidine (GAPH), and glycine-aspartic acid-alanine-histidine (GDAH) (Figure 2B). In addition, the sequence encoding all four amino acids, GDPH, was deleted, referred to as SUSD2-**∆**GDPH (see diagrams in Figure 1A and Figure S1).

293T cells were transiently transfected with expression plasmids encoding SUSD2-GDPH mutants, and whole cell lysates (WCL) were harvested for western immunoblot analysis of SUSD2 fragments. When glutamic acid was substituted for aspartic acid (GEPH), no inhibition of SUSD2 cleavage was observed (Figure 2B). Previously, Lidell et al. characterized a D to E mutation in MUC5AC. However, unlike SUSD2, the single amino acid change inhibited cleavage of MUC5AC [13]. Substituting alanine for proline (GDAH) resulted in only a slight inhibition of SUSD2 cleavage. However, substituting alanine for aspartic acid (GAPH) resulted in a large decrease in SUSD2 cleaved fragments and an enrichment of the precursor polypeptide (Figure 2B). Deletion of all four amino acids, SUSD2-**∆**GDPH, completely inhibited cleavage and resulted in production of only the precursor polypeptide (Figure 2B). In order to discern the biological significance of SUSD2 cleavage, we continued to study the noncleaved SUSD2-**∆**GDPH deletion mutant.

### 2.2. SUSD2 Cleavage is Required for its Cell Surface Localization and Surface Presentation of Gal-1

We have previously shown using flow cytometry analysis and immunofluorescence staining that SUSD2 localizes to the plasma membrane in MDA-MB-231-SUSD2 and SKBR3 cell lines [1]. Therefore, stable MDA-MB-231-SUSD2-**∆**GDPH cells were generated to characterize the cellular localization of SUSD2-**∆**GDPH. Flow cytometry analysis of MDA-MB-231-SUSD2-**∆**GDPH revealed that SUSD2-**∆**GDPH was unable to localize to the plasma membrane (Figure 2C). Western immunoblot analysis of stable MDA-MB-231-SUSD2-**∆**GDPH produced a 110 kDa SUSD2 band that lacked any cleaved fragments, indicating that SUSD2-**∆**GDPH is being produced, but it cannot translocate to the plasma membrane. These data suggest that SUSD2 cleavage is required for its proper localization at the cell surface.

Previously, our group demonstrated that Gal-1 requires SUSD2 to localize to the plasma membrane [1]. Therefore, we asked the question of whether cleavage of SUSD2 was required for Gal-1 surface presentation. Using the stable cell line MDA-MB-231-SUSD2-**∆**GDPH, flow cytometry analysis showed that Gal-1 surface presentation was lost, while MDA-MB-231-SUSD2 cells presented both SUSD2 and Gal-1 at the cell surface (Figure 2C). To confirm that SUSD2-**∆**GDPH is not presented at the cell surface, immunofluorescent staining for SUSD2 was performed. Stable MDA-MB-231-SUSD2 and MDA-MB-231-SUSD2-**∆**GDPH cells were stained for SUSD2 in both permeabilized and nonpermeabilized conditions. MDA-MB-231-SUSD2 cells stained positive for SUSD2 in both the permeablized and nonpermeablized conditions (Figure 2D). The MDA-MB-231-SUSD2-**∆**GDPH cells showed no staining for SUSD2 when cells were not permeablized. However, permeablized MDA-MB-231-SUSD2-**∆**GDPH cells showed SUSD2 staining with a perinuclear localization (Figure 2D). These staining patterns indicate that SUSD2-**∆**GDPH can no longer localize to the cell surface, but instead remain trapped inside the cell.

### 2.3. SUSD2 is Cleaved in the Endoplasmic Reticulum

**Figure 3:**
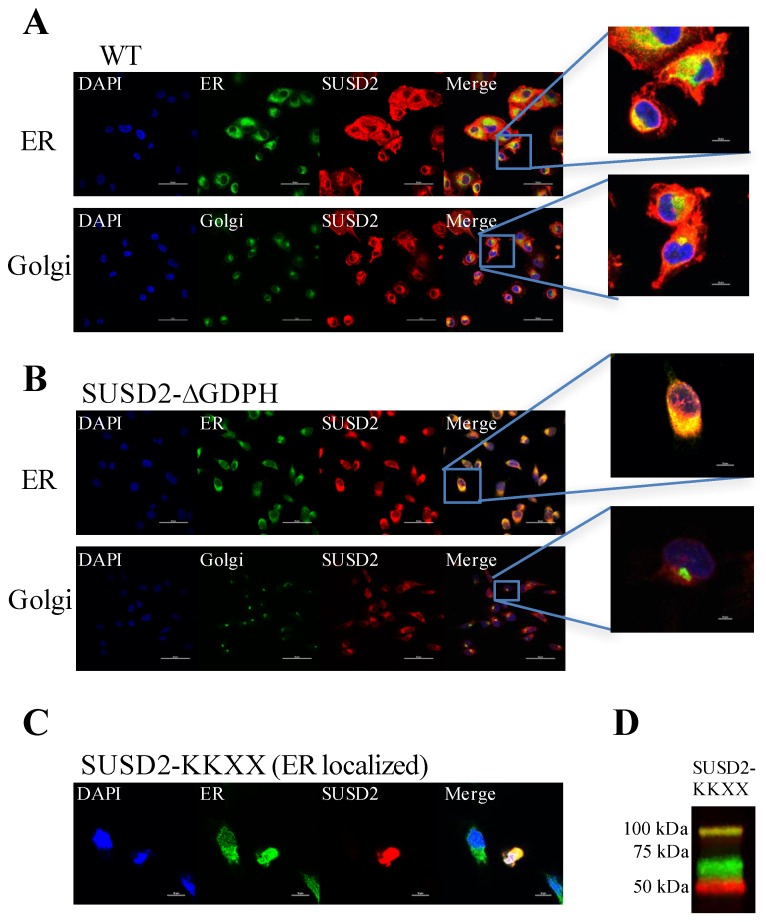
Immunofluorescence staining of stable MDA-MB-231-SUSD2 and stable MDA-MB-231-SUSD2-∆GDPH cells. Cells were grown on glass coverslips overnight. Prior to staining, cells were fixed with 2% paraformaldehyde and permeabilized with 0.2% Triton X-100. (**A**) MDA-MB-231-SUSD2 or (**B**) MDA-MB-231-SUSD2-∆GDPH cells were stained for SUSD2 (anti-SUSD2, red) and endoplasmic reticulum (ER) (anti-KDEL, green) (top panels), or for SUSD2 (anti-SUSD2, red) and Golgi (anti-58K Golgi, green) (bottom panels). Nuclei are stained blue using DAPI. (**C**) Effect of an ER retention signal on SUSD2 processing. MDA-MB-231 cells were transiently transfected with an expression construct encoding SUSD2 fused to an ER retention signal (SUSD2-KKXX). Cells were stained for both SUSD2 and ER using anti-SUSD2 (red) and anti-KDEL (green) antibodies to confirm ER localization of this mutant. (**D**) SUSD2-KKXX is cleaved in the ER. Western immunoblot analysis of 293T cells transiently transfected with pSUSD2-KKXX was performed. Cell lysates were separated by gel electrophoresis, transferred to a PVDF membrane, and probed with anti-N-terminal (red) and anti-C-terminal (green) SUSD2 antibodies. Yellow staining indicates the presence of the full-length polypeptide.

Since SUSD2 is a type 1 transmembrane protein, we hypothesized that noncleaved SUSD2-**∆**GDPH may be sequestered in an earlier part of the secretory pathway such as the endoplasmic reticulum (ER) or the Golgi. In order to identify the localization of noncleaved SUSD2, immunofluorescent confocal microscopy was utilized. Stable MDA-MB-231-SUSD2 and MDA-MB-231-SUSD2-**∆**GDPH cells were co-stained with anti-SUSD2 antibody (red) and either anti-KDEL antibody (green) or anti-58K Golgi protein antibody (green) to determine if noncleaved SUSD2-**∆**GDPH accumulated in the ER or the Golgi, respectively. WT SUSD2 was localized to the cell surface, and was shown to colocalize with the ER and Golgi (Figure 3A). However, SUSD2-**∆**GDPH showed strong colocalization with the ER and minimal-to-no colocalization with the Golgi (Figure 3B), suggesting that cleavage of SUSD2 takes place in the ER and may be necessary for its transit out of the ER. To assess if SUSD2 cleavage occurs in the ER, an expression construct was generated encoding SUSD2 with the ER transmembrane protein retention signal, KKXX, at the C-terminal end, referred to as pSUSD2-KKXX (Figure S1). Retention of SUSD2-KKXX in the ER was verified by confocal microscopy using MDA-MB-231 cells that were transiently transfected with pSUSD2-KKXX (Figure 3C). Western immunoblot analysis of this mutant in 293T cell lysates revealed that despite retention in the ER, SUSD2-KKXX was still cleaved (Figure 3D), which is consistent with previous data demonstrating that MUC5AC is cleaved at its GDPH site in the ER as well [13].

### 2.4. Pulse-Chase Analysis of SUSD2 Post-Translational Processing and Mechanism of Cleavage

Cleavage of GDPH sequences has been shown to occur via two distinct mechanisms, either through pH autocatalytic cleavage [14] or through the activity of a serine protease [15]. To define how SUSD2 is cleaved, both these mechanisms were investigated. MDA-MB-231-SUSD2-SNAP stable cell lines were generated to analyze the SUSD2-SNAP fusion protein and subsequent pulse-chase experiments. Western immunoblot analysis of SUSD2-SNAP showed the expected 19 kDa size increase in the C-terminal fragment and precursor. In addition, SUSD2-SNAP was still cleavable (Figure S3A). Flow cytometry showed that SUSD2-SNAP localized to the cell surface (Figure S3B). This indicated that adding the SNAP tag did not interfere with SUSD2 localization or cleavage. Pulse-chase methods are described in the Experimental Procedures Section. Pulse-chase analysis of SUSD2 production indicated that the SUSD2 precursor was present at 1 h and the cleaved band appeared between 1 and 4 h (Figure S3C).

To determine the effect of pH on SUSD2 cleavage, ammonium chloride was utilized to neutralize the secretory pathway. MDA-MB-231-SUSD2-SNAP cells were blocked with BTP and incubated with 25 mM ammonium chloride. After 6 h, lysates were harvested and labeled with SNAP-surface 682. Western blot visualization of fluorescence SNAP-surface 682 labeled SUSD2-SNAP showed that SUSD2 cleavage was not inhibited by pH neutralization (Figure S4A). Although low pH enhanced MUC5AC autocatalytic cleavage, MUC5AC was shown to undergo autocatalytic cleavage at the neutral pH of the ER [13]. Since MUC5AC was still cleaved at neutral pH, the ability of SUSD2 to undergo autocatalytic cleavage at neutral pH was tested. To discern the contribution of autocatalytic or proteolytic mechanisms of SUSD2 cleavage, a cell free system was utilized so that endogenous proteases would not be present to cleave SUSD2. Recombinant SUSD2 (IVT SUSD2) was generated by in vitro transcription/translation (see Experimental Procedures for details) using the 1-Step Human In Vitro Protein Expression Kit (ThermoFisher Scientific, Waltham, MA, USA). The reaction conditions for IVT SUSD2 production were carried out at neutral pH. Western immunoblot analysis of IVT SUSD2 with both anti-N- and anti-C-terminal antibodies demonstrated that IVT SUSD2 was not cleaved (Figure S4B).

**Figure 4:**
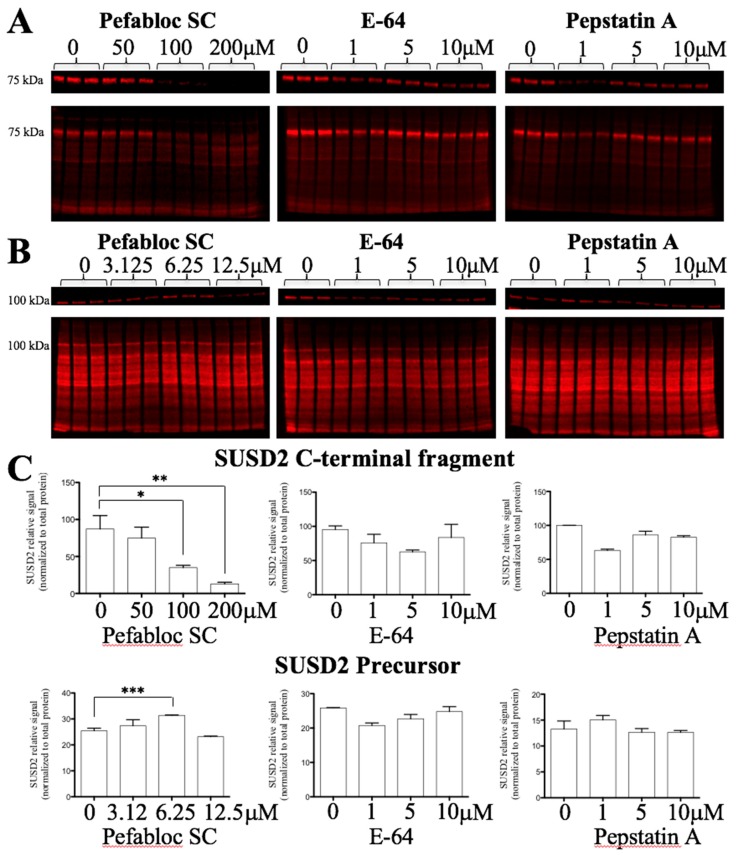
Fluorescence imaging of pulse-chase analysis (top) and REVERT total protein stain (bottom) of (**A**) SUSD2 C-terminal fragment or (**B**) SUSD2 precursor run in triplicate. MDA-MB-231-SUSD2-SNAP cells were treated with SNAP-Cell Block followed by incubation with Pefabloc SC for 24 (**A**) or 6 (**B**) hours. Lysates were harvested and labeled with SNAP-surface 682 fluorophore. Proteins were separated by gel electrophoresis, transferred to a membrane, and imaged using LI-COR Odyssey Fc Imager. After pulse-chase imaging, membranes were stained with REVERT total protein stain (LI-COR) and imaged using LI-COR Odyssey Fc Imager. (**C**) Pefabloc SC inhibits cleavage of SUSD2 and increases the amount of precursor. Quantification of results from (**A** and **B**) was performed by using the LI-COR Image Studio Software. * indicates *p*-value < 0.05, ** indicates *p*-value < 0.01, and *** indicates *p*-value < 0.001.

Since neutral pH did not induce autocatalytic cleavage, protease inhibitors were used to determine if SUSD2 is cleaved by a proteolytic mechanism. Pulse-chase analysis was performed on MDA-MB-231-SUSD2-SNAP cells treated with various protease inhibitors, including E-64 (cysteine), Pepstatin A (aspartic), and Pefabloc SC (serine). If cleavage was being inhibited, the bands representing the cleaved fragments should decrease over time while the levels of the precursor polypeptide should increase. Pefabloc SC significantly inhibited the accumulation of the cleaved SUSD2 fragments in a dose dependent manner compared to untreated controls at 24 h (Figure 4A). Two hundred µM Pefabloc SC showed a 75% decrease in the cleaved band with a *p*-value < 0.01 (Figure 4C). E-64 and Pepstatin A did not induce any dose dependent inhibition of SUSD2 cleavage (Figure 4A and quantification shown in Figure 4C). To determine the effect of Pefabloc SC on the SUSD2 precursor, lower doses were used at a 6-h time point. At 6 h, Pefabloc SC induced an increase in SUSD2 precursor at 3.125 and 6.25 µM. At 12.5 µM, the precursor levels dropped off (Figure 4B). E-64 and pepstatin did not induce any dose dependent change in the SUSD2 precursor (Figure 4B). Quantification of these western blots is shown in Figure 4C and indicates relative SUSD2 signal normalized to total protein. Pefabloc SC induced a significant increase in SUSD2 precursor at 6 h with a *p*-value < 0.001.

### 2.5. SUSD2 Fragments Remain Associated at the Cell Surface by Disulfide Bonds that are Required for SUSD2 Cleavage and Gal-1 Surface Presentation

**Figure 5:**
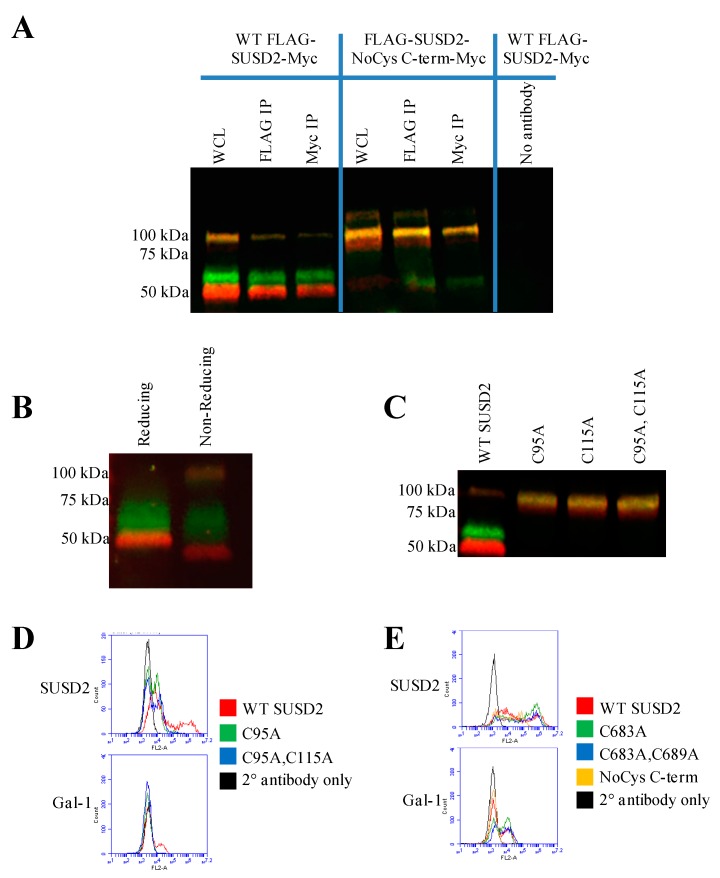
Immunoprecipitation and flow cytometry analysis of FLAG-SUSD2-Myc. (**A**) 293T cells were transfected with either pFLAG-SUSD2-myc or pFLAG-SUSD2-NoCys C-term-myc. Whole cell lysates (WCL) were immunoprecipitated with anti-FLAG or anti-Myc antibodies. Western immunoblot analysis using anti-N-terminal (red) and anti-C-terminal (green) SUSD2 antibodies was performed. Yellow indicates the presence of the full-length SUSD2 polypeptide. WCLs were used as a positive control. (**B**) SUSD2 precursor polypeptide present under nonreducing conditions. Western immunoblot analysis of SUSD2 from SKBR3 WCL was performed under reducing and nonreducing conditions. For nonreducing conditions, β-mercaptoethanol was omitted from the loading buffer. Antibodies used for western immunoblot analysis are the same as above in (**A**). (**C**) SUSD2 C-terminal cysteine to alanine mutations inhibited cleavage. Western immunoblot analysis was performed on 293T cells transiently transfected with plasmids encoding FLAG-SUSD2-Myc N-terminal single cysteine mutants, C95A and C115A, as well as the double mutant C95A and C115A. Anti-N-terminal (red) and anti-C-terminal (green) SUSD2 antibodies were used. Flow cytometry analysis of SUSD2 N-terminal (**D**) and C-terminal (**E**) cysteine mutants. 293T cells were transiently transfected with plasmids encoding WT SUSD2 or SUSD2 cysteine mutants as indicated. After 48 h, cells were labeled with anti-SUSD2 or anti-Gal-1 antibodies and analyzed for surface staining using the Accuri C6 flow cytometer. WT SUSD2 was used as a positive control, and 2° antibody was a negative control.

To determine whether the N- and C-terminal SUSD2 fragments remain associated after cleavage, immunoprecipitation (IP) was performed on pFLAG-SUSD2-Myc transiently transfected 293T protein lysates. Either anti-FLAG or anti-Myc antibodies were used for IP followed by western immunoblot analysis of SUSD2 (Figure 5A). The anti-FLAG antibody precipitated both the N- and C-terminal SUSD2 fragments, and reciprocal co-IP using anti-Myc antibody also precipitated both SUSD2 fragments (Figure 5A, left panel), which demonstrated that the fragments of human SUSD2 remain associated after cleavage. Consistently, western immunoblot analysis of SUSD2 under nonreducing conditions revealed a 110 kDa band that was not observed in reducing conditions (Figure 5B). The presence of the 110 kDa band under nonreducing conditions suggests that the SUSD2 fragments remain associated by one or more interchain disulfide bond(s).

To confirm that the two cleaved SUSD2 fragments are held together by disulfide bonds, site-directed mutagenesis was utilized to substitute cysteine with alanine (Figure 1A and Figure 3). The encoded alanine mutations of C-terminal cysteines 683 (C683A) and 689 (C689A) were generated individually and in combination in pFLAG-SUSD2-Myc. Constructs were transiently transfected into 293T cells followed by immunoprecipitation (IP) using either anti-FLAG or anti-Myc antibodies. Western immunoblot analysis showed that the N- and C-terminal fragments of FLAG-SUSD2 C683A and/or C689A-Myc were pulled down using either the anti-FLAG or anti-Myc antibodies, indicating that inter-fragment disulfide bonds were still being formed. Next, all six cysteines on the C-terminal fragment were substituted with alanine (SUSD2 NoCys C-term, Figure 1A and Figure 3), and the plasmid was transfected into 293T cells. IP using anti-FLAG or anti-Myc antibodies followed by western immunoblot analysis revealed a strong band representing the full-length polypeptide, indicating that the SUSD2 NoCys C-term protein was not cleaved. (Figure 5A, middle panel). This unexpected result suggested that inter-fragment disulfide bonding is required for SUSD2 cleavage to occur.

To determine the N-terminal cysteines that participate in inter-fragment disulfide bonds, cysteines 95 and 115 were selected for substitution with alanine because neither cysteine amino acid was located within a domain (Figure 1A). Expression plasmids with single and combined alanine mutations in pFLAG-SUSD2-Myc were generated (C95A, C115A, or C95A and C115A, Figure 1A and Figure S1). SUSD2 mutants containing C95A, C115A, or C95A and C115A could not be cleaved, as indicated by the prominent, full-length, yellow SUSD2 bands detected at 110 kDa (Figure 5C).

Next, we investigated whether the SUSD2 cysteine to alanine mutant proteins were able to localize to the plasma membrane and whether Gal-1 would be presented at the cell surface. Flow cytometry analysis was performed on nonpermeabilized cells such that only proteins on the cell surface were detected. Results for the N-terminal C95A, or C95A and C115A SUSD2 mutants demonstrated that SUSD2 cell surface localization (Figure 5D) was impaired when the protein was not cleaved (Figure 5C). Consistently, localization of Gal-1 on the cell surface correlated with the presence of SUSD2 on the plasma membrane (Figure 5D). Transient transfections of 293T cells with plasmids encoding the C-terminal mutations revealed that Gal-1 was localized to the cell surface when SUSD2 was cleavable, including SUSD2 C683A, C689A, as well as the double C-terminal change, C683A and C689A (Figure 5E). The NoCys C-term SUSD2 mutant was not cleaved (Figure 5A, middle panel), and neither NoCys C-term SUSD2 nor Gal-1 was localized to the cell surface (Figure 5E). The ability of the cysteine mutants to present Gal-1 on the cell surface was dependent on their ability to be cleaved, which is consistent with SUSD2-**∆**GDPH data which showed that SUSD2 cleavage was required for cell surface presentation of Gal-1 (Figure 2C).

**Figure 6:**
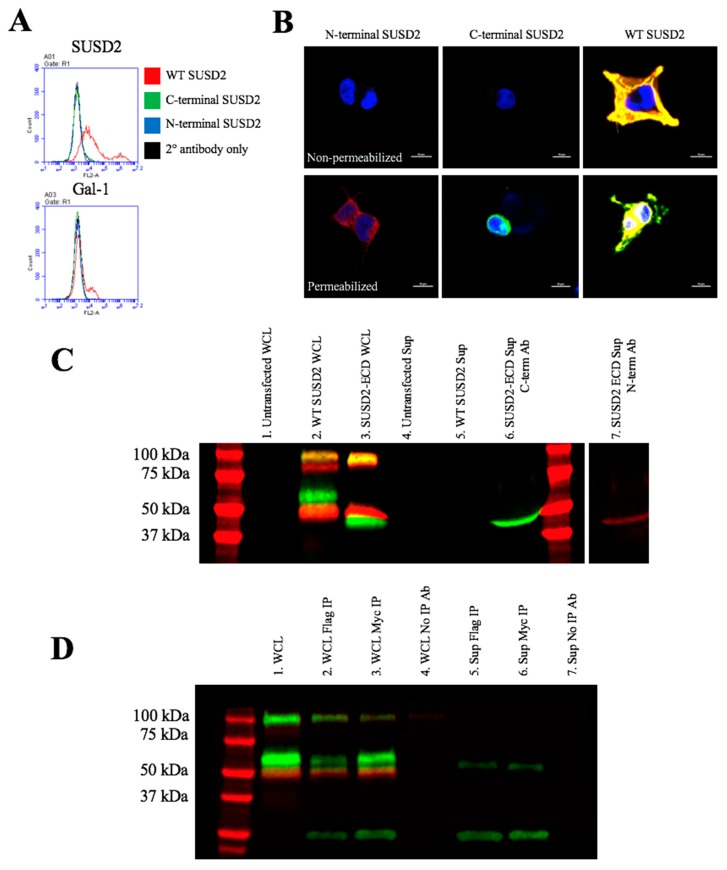
Flow cytometry and immunofluorescence analysis of N- and C-terminal fragments of SUSD2. (**A**) Flow cytometry analysis of N- and C-terminal SUSD2 fragments and Gal-1 plasma membrane localization. 293T cells were not permeabilized. The anti-N-terminal SUSD2 antibody was used to label 293T cells transiently transfected with N-terminal, C-terminal, or WT SUSD2. The anti-Gal-1 antibody was used to detect cell surface Gal-1. (**B**) Immunofluorescence confocal microscopy was utilized to determine the localization of N- and C-terminal fragments. Cells were fixed with 2% paraformaldehyde and permeabilized with 0.2% Triton X-100 before staining with anti-N-terminal (red) or anti-C-terminal (green) SUSD2 antibodies. Yellow indicates the colocalization of both fragments. (**C**) Western immunoblot analysis of SUSD2 in cell supernatants. 293T cells were transiently transfected with expression constructs encoding WT SUSD2 or SUSD2-ECD. The SUSD2-ECD is a positive control for secretion. Supernatants and whole cell lysates (WCL) were harvested 48-h post-transfection and analyzed by western immunoblot. Supernatants and WCL were separated by SDS-PAGE and probed for SUSD2 using anti-C-terminal (green) and anti-N-terminal (red) SUSD2 antibodies. (**D**) Immunoprecipitation analysis of FLAG-SUSD2-Myc in cell supernatants. 293T cells were transiently transfected with pFLAG-SUSD2-Myc. After 48 h, WCL and supernatants were harvested. Immunoprecipitation was performed using anti-FLAG or anti-Myc antibodies. Immunoprecipitated proteins from supernatants and WCL were separated by SDS-PAGE and probed for SUSD2 using anti-C-terminal (green) and anti-N-terminal (red) SUSD2 antibodies.

Since disulfide bonding of the SUSD2 fragments was necessary for cleavage and surface localization, we hypothesized that the mature form of SUSD2 is a disulfide bonded heterodimer of the N- and C-terminal fragments, and that both fragments would be required for surface presentation of Gal-1. To confirm this, we generated expression plasmids that encoded either the N- or C-terminal fragment of SUSD2 as defined by the GDPH cleavage sequence. Plasmids encoding the fragments were transiently transfected alone into 293T cells for analysis of their ability to present Gal-1 independently of the other fragment. Flow cytometry analysis revealed that neither the N- nor C-terminal fragment alone could present Gal-1 at the cell surface (Figure 6A). To verify that the SUSD2 fragments were being produced in the cells and determine their cellular localization, immunofluorescence confocal microscopy was performed (Figure 6B). Under nonpermeabilized conditions, neither the N- nor C-terminal SUSD2 fragments were localized to the cell surface (Figure 6B, top panels). However, staining the SUSD2 N- and C-terminal fragments using permeabilized conditions (Figure 6B, bottom panels) revealed a perinuclear localization similar to SUSD2-**∆**GDPH (Figure 2D). As a positive control, confocal microscopy of 293T cells transfected with WT SUSD2 showed strong colocalization of the fragments at the cell surface as indicated by the yellow color (Figure 6B).

### 2.6. SUSD2 Remains on the Cell Surface and is not Secreted

The N-terminal fragment of MUC4 is secreted after its cleavage at the GDPH site [15,16]. In addition, MUC4 has an alternative splice form, which removes the transmembrane domain causing the extracellular domain of the protein to be secreted [17]. Since SUSD2 is a paralog to MUC4, we investigated whether SUSD2 was secreted. As a positive control for secretion, pSUSD2-ECD was utilized (Figure S1). Supernatants and cell lysates were collected from 293T cells transiently transfected by plasmids encoding SUSD2-ECD or WT SUSD2, and western immunoblot analysis using anti-N-terminal and anti-C-terminal SUSD2 antibodies was performed (Figure 6C). Whole cell lysates of cells containing WT SUSD2 or SUSD2-ECD (Figure 6C, lanes 2 and 3, respectively) showed that both N- and C-terminal fragments of SUSD2 were being produced. The C-terminal fragment (green) of SUSD2-ECD is smaller than that of the WT because the transmembrane domain located at the C-terminal end of the protein has been removed. However, neither the N- nor the C-terminal fragments of WT SUSD2 were detected in the supernatant (Figure 6C, lane 5), suggesting that the WT SUSD2 C-terminal and N-terminal fragments remain membrane bound and are not secreted (Figure 6C). Both the C- and N-terminal fragments were detected for the secreted control, SUSD2-ECD (Figure 6C, lanes 6 and 7, respectively). The C-terminal and N-terminal bands of SUSD2-ECD were imaged separately to ensure the presence of both bands since they are of similar size and more dilute in the supernatants compared to whole cell lysates.

To support this conclusion, we performed immunoprecipitation on whole cell lysates and supernatants derived from 293T cells transiently transfected with pFLAG-SUSD2-Myc using anti-FLAG or anti-Myc antibodies (Figure 6D). Both SUSD2 fragments, as well as the uncleaved precursor, were present in whole cell lysates (Figure 6D, lane 1), and all three fragments were pulled-down using anti-FLAG or anti-Myc antibodies (Figure 6D, lanes 2 and 3, respectively). Interestingly, neither the N- nor C-terminal fragments of FLAG-SUSD2-Myc were detected from supernatants after IP with anti-FLAG or anti-Myc antibodies (Figure 6D, lanes 5 and 6). The green bands present at 25 and 50 kDa are the heavy and light chain fragments of the anti-FLAG and anti-Myc antibodies used for IP. Both IP antibodies, as well as the anti-C-terminal SUSD2 antibody, were generated in mice and are recognized by the anti-mouse 800CW conjugated secondary antibody. Taken together, these results suggest that neither the N- nor C-terminal WT SUSD2 fragments are secreted, and that both fragments remain at the cell surface, tethered by disulfide bonds. By immunoprecipitation analysis, SUSD2 was not present in the cell supernatant. In the future, a sandwich ELISA will be performed for a more precise and absolute quantification of SUSD2 in the supernatant.

## 3. Discussion

SUSD2 is a paralog to MUC4 and contains a VWFD domain which is conserved in many MUC family members [13,14,15]. Identical to MUC4, our data demonstrate that SUSD2 is cleaved between the aspartic acid and proline residues of the GDPH sequence in the VWFD domain (Figure 1A and Figure 2B). Although several MUC family members are cleaved at the conserved GDPH site, the cleavage may have different functional consequences depending on the specific protein. A summary of the similarities and differences of SUSD2, MUC4, MUC5AC, and MUC2 processing can be found in Table 1. For example, when cleavage of the GDPH site in MUC5AC was inhibited, the uncleaved MUC5AC protein traveled through the secretory pathway [13]. However, when SUSD2 GDPH cleavage was inhibited, SUSD2 was sequestered in the ER and was unable to traffic to the plasma membrane (Figure 4A and Figure 5B). Similar to SUSD2, MUC4-GDPH cleavage was required for its secretory pathway trafficking, as precursor MUC4 was degraded if it could not be cleaved [18].

SUSD2 shares the GDPH sequence in common with MUC4, MUC5AC, and MUC2. Each of these proteins is cleaved at its GDPH sequence. However, the consequences of this cleavage are different in these proteins. Table 1 highlights notable similarities and differences between SUSD2 and three MUC Family proteins. References are shown in parentheses.

Cleavage in the GDPH sequence has been shown to occur in multiple proteins and by two distinct mechanisms, autocatalytic- and protease-dependent [13,14,15,21,22]. The mechanism of MUC5AC cleavage was a pH dependent autolysis at the GDPH sequence. This type of autocatalytic cleavage between aspartic acid and proline is proposed to operate through generation of an unstable five-membered ring intermediate [22]. This cleavage was abolished by mutagenesis of the GDPH sequence to GEPH in MUC5AC, which would prevent formation of this intermediate [13]. Interestingly, cleavage of SUSD2-GEPH was not impaired (Figure 2B). This mutation did not inhibit SUSD2 cleavage, suggesting that SUSD2 cleavage may not be dependent on an autocatalytic mechanism like MUC5AC. To further support a nonautocatalytic cleavage, in vitro transcription/translation was used to produce IVT SUSD2. Unlike the processing of SUSD2 in mammalian cells (Figure 1B), IVT SUSD2 was not cleaved (Figure S3B). These data are more consistent with an enzymatic mechanism of cleavage than an autocatalytic mechanism.

MUC4 is cleaved at its GDPH sequence through a proteolytic mechanism involving an unknown serine protease [15]. Since MUC4 is a paralog of SUSD2, we hypothesized that SUSD2 may have the same cleavage mechanism as MUC4. Treating stable MDA-MB-231-SUSD2-SNAP cells for 24 h with Pefabloc SC, a pan serine protease inhibitor, demonstrated a significant dose dependent decrease in the SUSD2 C-terminal fragment (Figure 4A,C). One would predict that inhibition of SUSD2 cleavage would result in the accumulation of the precursor polypeptide as amounts of cleaved fragments decreased. However, we found that at 50–200 µM concentrations of Pefabloc SC, SUSD2 full-length precursor levels decreased. The disappearance of the precursor SUSD2 at these higher doses can likely be attributed to the degradation of unprocessed SUSD2 as observed with MUC4 precursor [18]. To identify an initial increase in the precursor before its degradation, we analyzed SUSD2 precursor levels at lower doses of Pefabloc SC and at an earlier time point (6 h). We observed a significant increase in SUSD2 precursor over 6 h at 6.25 µM Pefabloc SC, while E-64 and pepstatin A did not affect SUSD2 precursor levels significantly (Figure 4B,C).

Reciprocal IP of dual tagged FLAG-SUSD2-Myc confirmed that the SUSD2 fragments remained associated after cleavage and that neither fragment was secreted into the supernatant (Figure 6). Other groups have demonstrated that the secretion of MUC2 and MUC5AC does not require GDPH cleavage [19,20]. In contrast, MUC4 must be cleaved for the N-terminal fragment to be secreted into the extracellular environment [16]. Western immunoblot analysis in nonreducing conditions suggested that the SUSD2 fragments remained associated by at least one inter-fragment disulfide bond (Figure 5B). Mutagenesis of targeted cysteines on SUSD2 revealed that inter-fragment disulfide bonding is required in order for SUSD2 to be cleaved (Figure 5E).

SUSD2 contains two N-terminal (C95, C115) and two C-terminal (C683, C689) cysteines that are located outside a defined domain. When both sets of cysteines were substituted with alanine alone or in combination, C95A, C115A, or both combined, a complete inhibition of SUSD2 cleavage was observed (Figure 5C), suggesting that both amino acids C95 and C115 participate in inter-fragment disulfide bonds and are required for cleavage of SUSD2. Consistent with previous data, C95A, C115A, as well as combined C95A and C115A SUSD2 mutants were unable to present Gal-1 at the cell surface (Figure 5D). Since disulfide bonding of SUSD2 fragments was critical for its cleavage, we hypothesized that the mature form of SUSD2 is composed of both fragments. As expected, neither the C- nor N-terminal SUSD2 fragments alone were able to present Gal-1 at the cell surface as analyzed by flow cytometry (Figure 6A). Confocal microscopy analysis utilizing C-terminal and N-terminal specific antibodies further demonstrated that fragments expressed alone were localized in a perinuclear pattern similar to SUSD2-∆GDPH (Figure 2D and Figure 6B). However, WT SUSD2 fragments colocalized strongly at the cell surface in nonpermeabilized cells and throughout the cell in permeabilized conditions (Figure 6B).

Immunofluorescence staining of WT SUSD2 confirmed surface localization and colocalization with ER and Golgi markers (Figure 3A). These results indicate that WT SUSD2 is found in the traditional secretory pathway. However, SUSD2-∆GDPH is not found at the cell surface, and it colocalizes with the ER marker but not Golgi marker (Figure 3B), suggesting that SUSD2 cleavage occurs in the ER and appears to be necessary for SUSD2 to transit out of the ER. In addition, SUSD2-KKXX was cleaved while being retained in the ER (Figure 3C,D).

Taken together, our results indicate that SUSD2 is cleaved at the GDPH site in the ER by a yet unidentified serine protease. The SUSD2 N- and C-terminal fragments are both critical for surface localization of SUSD2 and Gal-1. SUSD2 must form disulfide bonds before cleavage and is transported to the membrane as a heterodimer with the fragments connected via disulfide bonds. SUSD2 chaperones Gal-1 to the cell surface of breast cancer cells, which has potential implications for immune evasion in breast cancer. Discovery of the identity of this protease could prove useful in targeting SUSD2 production as a therapeutic intervention in breast cancer by preventing Gal-1 surface presentation.

## 4. Materials and Methods

### 4.1. Design of SUSD2 Mutant Plasmids

SUSD2 mutants were generated using the Quikchange XL site-directed mutagenesis kit (Agilent Technologies, Santa Clara, CA, USA). The pcDNA3.1-myc/his containing wild-type *SUSD2* was generated previously [1] and used as the template for mutagenesis. The SUSD2 extracellular domain (SUSD2-ECD) was generated by inserting a stop codon directly before the transmembrane domain. Primers were synthesized by IDT, Coralville, IA, USA and shown in Table S1. The expression construct encoding SUSD2 with the ER retention signal, KKXX, at the C-terminal end, referred to as pSUSD2-KKXX, was generated by inserting two lysine codons at positions immediately 5′ to the final 2 histidine codons of SUSD2 myc/his. This strategy generated a SUSD2 protein with lysines at the -3 and -4 position from the C-terminus.

SUSD2-GDPH mutants included SUSD2-GDPH deletion (SUSD2-∆GDPH), SUSD2-GEPH, SUSD2-GDAH, and SUSD2-GAPH. SUSD2-GDPH deletion primers were also used for mutagenesis with pLXSN-SUSD2 as a template. pLXSN-SUSD2 was generated previously by inserting the open reading frame of SUSD2 into the pLXSN plasmid without any tags [1]. pLXSN-SUSD2-∆GDPH was used to generate stable cell lines expressing *SUSD2-∆GDPH* without the myc/his tag.

SUSD2 cysteine mutants were generated by mutagenesis using pFLAG-SUSD2-Myc as a template. SUSD2 cysteine mutants included single mutations: C683A, C689A, C95A, and C115A; double mutations: C683A and C689A and C95A and C115A; and mutation of all six C-terminal fragment cysteines to alanine: C683A, C689A, C725A, C751A, C765A, and C778A. Mutagenesis primers are shown in Table S1.

The expression plasmid encoding the SUSD2-SNAP fusion protein was generated by inserting SUSD2 into the pSNAPf plasmid (NEB, Ipswich, MA, USA) using the *Eco*RV and *Eco*RI cloning sites. Genscript’s Gene Synthesis service generated pFLAG-SUSD2-Myc in pcDNA3.1 with the FLAG sequence on the 5′ end of *SUSD2* and myc sequence 3′ of *SUSD2*. The plasmid encoding the SUSD2 C-terminal fragment was synthesized by Genscript (Piscataway, NJ, USA), and the plasmid encoding the SUSD2 N-terminal fragment was generated using site-directed mutagenesis to insert a stop codon (TAA) directly 5′ of the nucleotides encoding the PH of the GDPH sequence. All plasmids generated were sequence verified using Simple Seq kit per manufacturer instructions (Eurofins Genomics, Louisville, Kentucky, USA). Sequences were analyzed using Sequencher DNA sequence analysis software (Gene Codes Corporation, Ann Arbor, Michigan, USA).

### 4.2. Cell Lines

MDA-MB-231, SKBR3, and 293T cells were maintained in DMEM supplemented with 10% fetal bovine serum (FBS) and penicillin and streptomycin at 37 °C in 5% CO_2_ in a humidified air incubator. Stable MDA-MB-231-SUSD2 cells have been described previously [1]. Stable MDA-MB-231-SUSD2 GDPH deletion (MDA-MB-231-SUSD2-∆GDPH) cells were generated using retroviral packaging Phoenix cells and pLXSN SUSD2-∆GDPH. Viral particles were used to infect MDA-MB-231 wild-type cells. Cells expressing SUSD2-∆GDPH were selected using 500 µM G418. Two single clones were isolated and designated MDA-MB-231-SUSD2-∆GDPH 1 and 9. Data shown in this paper are from MDA-MB-231-SUSD2-∆GDPH 1, and data are representative of results from both clones. MDA-MB-231-SUSD2-SNAP stable cells were generated by transfection with pSNAPf-SUSD2 and selection with G418. A clonal population was isolated and used for SUSD2-SNAP labeling assays. All cell lines were authenticated and tested negatively for mycoplasma.

### 4.3. Western Immunoblot Analysis

Western immunoblot analysis was performed as previously described [1] with modifications. Following gel electrophoresis, proteins were transferred to Immobilon-FL polyvinylidene fluoride (PVDF) membranes (EMD Millipore, Billerica, MA, USA). Membranes were allowed to dry completely after transfer. Methanol was used to rehydrate membranes prior to sequential washing with Tris buffered saline (TBS) and blocking with 10% blocking buffer in TBS. Primary antibodies against the SUSD2 N-terminus and C-terminus were incubated on the same membrane overnight at 4 °C. The N-terminal specific primary is a rabbit monoclonal antibody (Abcam, Cambridge, UK), and the C-terminal specific primary is a mouse monoclonal antibody clone 944812 (R&D Systems, Minneapolis, MN, USA). Membranes were washed and blocked again with 5% blocking solution in TBS. IRdye 680RD (red) anti-rabbit secondary and IRdye 800 (green) anti-mouse secondary were used to detect the N-terminal and C-terminal specific primaries, respectively. (LI-COR, Lincoln, NE, USA). Membranes were dried and then imaged using an Odyssey Fc imaging system. (LI-COR)

### 4.4. Edman Sequencing

Expi293 cells have been adapted to higher density culture and optimized for high production of proteins [23]. To facilitate secretion of SUSD2-ECD into the supernatant, an expression plasmid encoding the SUSD2 extracellular domain (pSUSD2-ECD) was designed by inserting a stop codon immediately prior to the transmembrane domain (diagram in Figure S1). Supernatant of transfected Expi293 cells were harvested one week after transfection. Duplicate gels were run, and protein from each gel was transferred to a separate PVDF membrane. The first membrane was probed using a mouse monoclonal C-terminal specific SUSD2 antibody clone #944812 (R&D Systems) and detected using an anti-mouse secondary antibody conjugated to alkaline phosphatase (Figure S1). Bands were visualized using colorimetric detection by addition of NBT/BCIP. This blot served as a reference that allowed the identification of the C-terminal band on the second membrane. The second membrane was stained with Coomassie to visualize the band that would be subjected to Edman degradation (Figure S2). The C-terminal band was harvested in sample buffer containing 5% β-mercaptoethanol from the Coomassie stained membrane and sequenced by Edman degradation at the UC Davis Molecular Structure Facility, Davis, CA, USA.

### 4.5. Flow Cytometry

Cells were harvested using trypsin and resuspended in phosphate buffered saline (PBS) containing 1% FBS and 0.1% NaN3. Surface staining of SUSD2 and Gal-1 was performed using primary antibodies against SUSD2 (Abcam) or Gal-1 (R&D Systems) with nonpermeabilized cells. The antibody to SUSD2 was generated using an immunogen composed of amino acids 544–691 of human SUSD2, corresponding to sequence from the N-terminal fragment of SUSD2. Excess primary antibody was washed away, and cells were incubated with PE conjugated secondary antibodies. Cells were analyzed using an Accuri C6 flow cytometer (BD Biosciences, San Jose, CA, USA).

### 4.6. Immunofluorescence Microscopy

Immunofluorescence microscopy was performed as previously described [1]. Triton X-100 was omitted in nonpermeabilized conditions. SUSD2 staining was performed using an N-terminal specific rabbit anti-SUSD2 antibody (Abcam) or a C-terminal specific mouse anti-SUSD2 antibody (Abnova, Taoyuan City, Taiwan). Mouse monoclonal anti-KDEL and mouse monoclonal anti-58K Golgi protein antibodies were used to visualize the endoplasmic reticulum (ER) and Golgi, respectively (Abcam). Slides were imaged using a Nikon A1 confocal microscope and analyzed using NIS elements software (Nikon, Melville, NY, USA).

### 4.7. Immunoprecipitation

Cell lysates were incubated with either M2 anti-FLAG (Sigma-Aldrich Corp., St Louis, MO, USA) or 4A6 anti-Myc (EMD Millipore) antibodies overnight at 4 °C on a rocking platform. Immune complexes were incubated with PureProteome Protein G magnetic beads (EMD Millipore) for 10 min at room temperature with constant inversion. Beads were then washed three times with 0.1% Tween 20 in PBS. Beads were separated from supernatant using a magnetic separator (EMD Millipore). Immune complexes were then boiled off the beads.

### 4.8. Fluorescent Pulse-chase

Stable MDA-MB-231-SUSD2-SNAP cells were utilized for fluorescent labeling of the SUSD2-SNAP fusion protein [24]. Cells were treated with 0.5 µM bromothenylpteridine (BTP) for 30 min, which blocks any previously produced SUSD2-SNAP fusion protein. Cells were washed 3 times with complete media and placed in the incubator until harvest at the appropriate time point. Lysates were labeled with 10 µM SNAP-Surface 682 in PBS with 1mM dithiothreitol (DTT), separated by gel electrophoresis, and proteins were transferred to a Immobilon-FL PVDF membrane (EMD Millipore). Membranes were imaged using LI-COR Odyssey Fc imager.

To assay the effect of protease inhibitors and pH on SUSD2 cleavage, fluorescence pulse chase was utilized. MDA-MB-231-SUSD2-SNAP cells were blocked with BTP as described above. After the final wash step to remove BTP, protease inhibitors were added to assess the effects of inhibitors on nascent SUSD2 only. MDA-MB-231-SUSD2-SNAP cells were treated with various protease inhibitors for 6 or 24 h. 1, 5, and 10 µM Pepstatin A (aspartic) (ThermoFisher Scientific), 3.125, 6.25, and 12.5 µM Pefabloc SC (serine) (Sigma-Aldrich Corp.) and 1, 5, and 10 µM E-64 (cysteine) (Sigma-Aldrich Corp.) were used separately to inhibit different classes of proteases. For pH neutralization experiments, complete media with 25 mM ammonium chloride was added to MDA-MB-231-SUSD2-SNAP cells. The pH was neutralized directly following the final wash step to remove BTP in order to visualize the effect of pH on only nascent SUSD2. After 6 h of protease inhibitor treatment or pH neutralization, cell lysates were harvested and labeled with 10 µM SNAP-surface 682 in PBS with 1 mM DTT for one hour at 37 °C. Following labeling, lysates were heated at 95 °C for 3 min and separated by gel electrophoresis before transfer to a PVDF membrane. The membrane was imaged using LI-COR Odyssey Fc imager. Subsequently, membranes were stained for total protein as a loading control using REVERT total protein stain per manufacturer instructions (LI-COR) and imaged again using LI-COR Odyssey Fc imager.

### 4.9. In Vitro Transcription/Translation of SUSD2

*In vitro* transcription/translation (IVT) of SUSD2 was produced using the 1-Step Human High-Yield Mini IVT Kit (ThermoFisher). SUSD2 was cloned into the pT7CFE1-NHis-GST vector using *Eco*RI and *Eco*RV restriction sites. IVT of SUSD2 was carried out according to manufacturer instructions.

## 5. Conclusions

Sushi Domain Containing 2 (SUSD2) is a type I transmembrane protein that is required for Gal-1 cell surface presentation. SUSD2 is cleaved at the GD-PH sequence in the endoplasmic reticulum. Our data suggests that SUSD2 cleavage is likely mediated by a serine protease. The inhibition of SUSD2 cleavage prevents SUSD2 and Gal-1 cell surface localization, indicating that SUSD2 cleavage is a maturation step in SUSD2 processing. We found that SUSD2 cleavage was dependent on the ability of SUSD2 to form inter-fragment disulfide bonds, which presumably form before cleavage. Once SUSD2 is cleaved, the fragments remain associated at the cell surface, with neither fragment being secreted into the extracellular space. We propose that mature, cleaved SUSD2 acts as a co-transporter for Gal-1 cell surface presentation, and disruption of SUSD2 disulfide bonding or cleavage inhibits Gal-1 presentation at the cell surface. Future studies of interest include identification and inhibition of the serine protease that cleaves SUSD2 as well as identification of strategies to selectively modify disulfide bond formation. Targeting SUSD2’s post-translational processing steps may provide novel mechanisms for combating the pro-tumor effects of SUSD2 and Gal-1 in breast cancer.

## Figures and Tables

**Table 1:** SUSD2 and MUC Family protein processing.

	SUSD2	MUC4	MUC5AC	MUC2
GDPH cleavage	† (Figure 2)	† [15]	† [13]	† [14]
Cleavage inhibition disrupts processing	† (Figure 2D and Figure 3)	† [18]	− [13]	− [14]
pH dependence	− (Figure S4)	− [15]	†/− [13]	† [14]
Protease dependence	† (Figure 4)	† [15]	− [13]	− [14]
Secreted	− (Figure 6C,D)	† [16]	† [19]	† [20]

## Supplementary Materials

Supplementary materials can be found at https://www.mdpi.com/1422-0067/20/15/3814/s1.
