# Supplementary material for: SUSD2 Proteolytic Cleavage Requires the GDPH Sequence and Inter-Fragment Disulfide Bonds for Surface Presentation of Galectin-1 on Breast Cancer Cells
Source: Int J Mol Sci. 2019 Aug 5;20(15):3814. doi: 10.3390/ijms20153814 (PMC6696261; doi:10.3390/ijms20153814)
Supplement: Supplementary file 1 [file ijms-20-03814-s001.pdf]

Supplementary Materials:

Table S1. Sequence of primers used for *SUSD2* mutagenesis.

Constructs	Primer Sequences
SUSD2-ECD	For 5′-GGACGCAGCTACGCGTAAGTGCTGTTGGGCATC-3′<br>Rev 5′-GATGCCCAACAGCACTTACGCGTAGCTGCGTCC-3′
SUSD2 ER	For 5′-CCGGTCATCATCACCATAAAAAGCACCATTGAGTTTAAACC-3′<br>Rev 5′-GGTTTAAACTCAATGGTGCTTTTTATGGTGATGATGACCGG-3′
SUSD2-ΔGDPH	For 5′-CTGGCCTCCGCCTTCTTTGTGACCTTCGAC-3′<br>Rev 5′-GTCGAAGGTCACAAAGAAGGCGGAGGCCAG-3′,
SUSD2-GEPH	For 5′-CTCCGCCTTCGGAGAGCCACACTTTGT-GACC-3′<br>Rev 5′-GGTCACAAAGTGTGGCTCTCCGAAGGCGGAG-3′
SUSD2-GDAH	For 5′-CTCCGCCTTCGGAGACGCACACTTTGTGACC-3′,<br>Rev 5′-GGTCACAAAGTGTGCGTCTCCGAAGGCGGAG-3′
SUSD2-GAPH	For 5′-CCTCCGCCTTCGGAGCCCCACACTTTGTGAC-3′<br>Rev 5′-GTCACAAAGTGTGGGGCTCCGAAGGCGGAGG-3′
SUSD2-C95A	For 5′-GACGCCAGTGTGATCGCCAGGTTTAAGGACAG-3′<br>Rev 5′-CTGTCCTTAAACCTGGCGATCACACTGGCGTC-3′,
SUSD2-C115A	For 5′-CTCCGGGCAAGTGCACGCTGTGTCACCTCTGCTC-3′<br>Rev 5′-GAGCAGAGGTGACACAGCGTGCACTTGCCCGG-AG-3′,
SUSD2-C683A	For 5′-GAGGCAGCCAAACTAGCTGGGGACGATCATTTC-3′<br>Rev 5′-GAAATGATCGTCCCCAGCTAGTTTGGCTGCCTC-3′,
SUSD2-C689A	For 5′-GGGGACGATCATTTCGCCAACTTTGATGTGGC-3′<br>Rev 5′-GCCACATCAAAGTTGGCGAAATGATCGTCCCC-3′
SUSD2-C725A	For 5′-CAGCCAGTGGTGTCCGCTGGCTGGCTGGCCCC-3′;<br>Rev 5′-GGGGCCAGCCAGCCAGCGGACACCACTGGCTG-3′
SUSD2-C751A	For 5′-CACCATCTACTTCCACGCTGACAACGGCTACAGC-3′<br>Rev 5′-GCTGTAGCCGTTGTCAGCGTGGAAGTAGATGGTG-3′
SUSD2-C765A	For 5′-GCAGAGACCAGCACCGCCCAGGCTGACGGCAC-3′<br>Rev 5′-GTGCCGTCAGCCTGGGCGGTGCTGGTCTCTGC-3′
SUSD2-C778A	For 5′-CTCACCCACCCCGAAGGCCCAGCCAGGACGCAGC-3′<br>Rev 5′-GCTGCGTCCTGGCTGGGCCTTCGGGGTGGGTGAG-3′

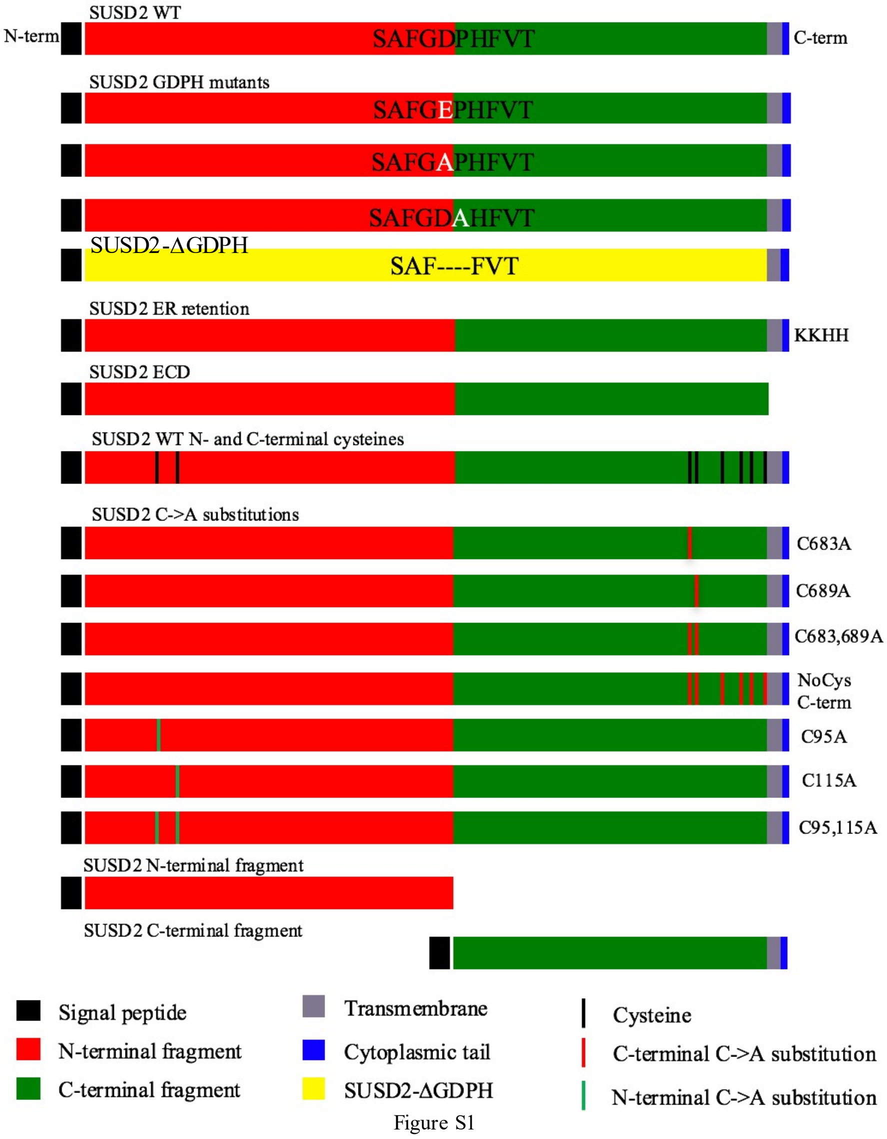

Figure S1. Diagram of SUSD2 mutant constructs. Shown is a representation of the SUSD2 protein encoded by the mutant plasmids used in this study. Regions are shown in proportion to the length of their protein sequence. The red, green, and yellow colors represent fluorescence of the anti-N- and anti-C-terminal SUSD2 antibodies used with the LI-COR imaging platform. All mutants were generated using site-directed mutagenesis with the exception of the C-terminal SUSD2 fragment, which was synthesized by Genscript. *pFLAG-SUSD2-myc* was also generated by Genscript and was the template for mutagenesis that generated the SUSD2 cysteine mutants.

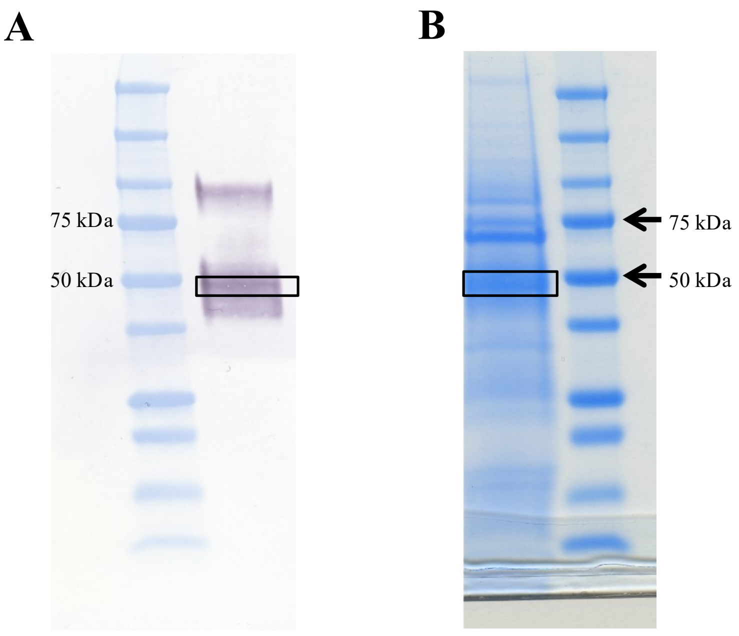

Figure S2

Figure S2. Isolation of SUSD2 C-terminal fragment for Edman sequencing. Expi293 cells were transiently transfected with pSUSD2-ECD (extra-cellular domain). Supernatants were harvested, separated by SDS-PAGE and transferred to PVDF membranes. (**A**) Western immunoblot analysis of SUSD2 extracellular domain using an anti-C-terminal SUSD2 antibody. (**B**) PVDF membrane stained with Coomassie Brilliant Blue. The band for Edman sequencing was harvested from this membrane.

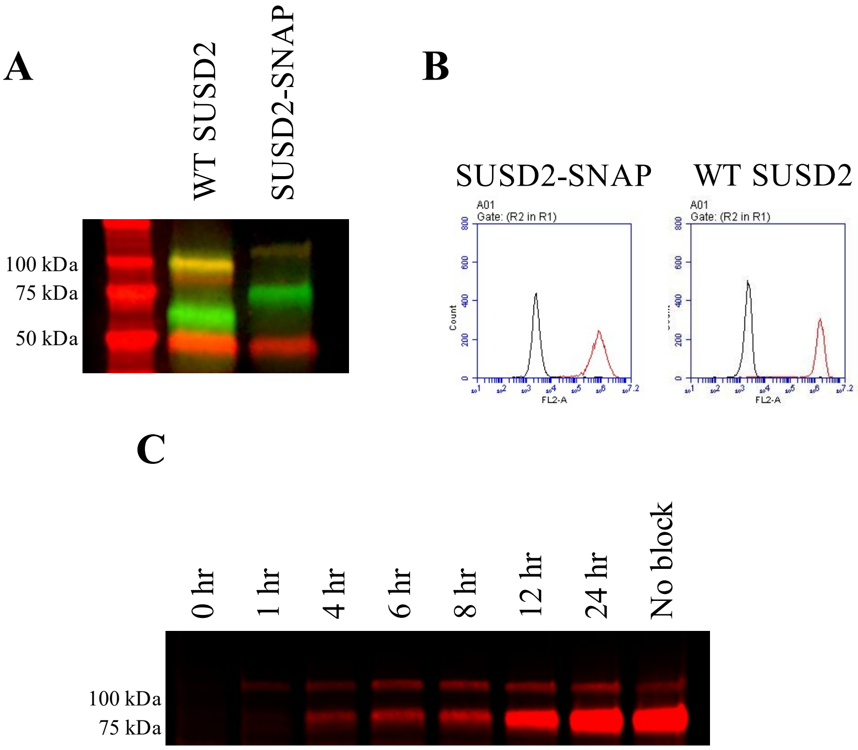

Figure S3

Figure S3. Characterization of SUSD2-SNAP fusion and pulse-chase analysis of SUSD2-SNAP production. (**A**) SUSD2-SNAP was cleaved similarly to WT SUSD2. 293T cells were transiently transfected with p*SUSD2-SNAP* and analyzed by western immunoblot analysis using anti-N-terminal (red) and anti-C-terminal (green) SUSD2 antibodies. (**B**) SUSD2-SNAP localized to the cell surface. Stable MDA-MB-231-SUSD2-SNAP cells were analyzed by flow cytometry for cell surface localization of SUSD2-SNAP. The red line indicates cell surface SUSD2 or SUSD2-SNAP and the black line is the 2° antibody negative control. (**C**) Pulse-chase analysis of SUSD2 production. Stable MDA-MB-231-SUSD2-SNAP cells were blocked with bromothenylpteridine (BTP) for 30 min followed by incubation to allow production of nascent SUSD2. A dish of cells was harvested at each indicated time point. The derived cell lysates were labeled with SNAP-surface 682 and separated by SDS-PAGE, transferred to PVDF membrane and imaged using LI-COR Odyssey Fc.

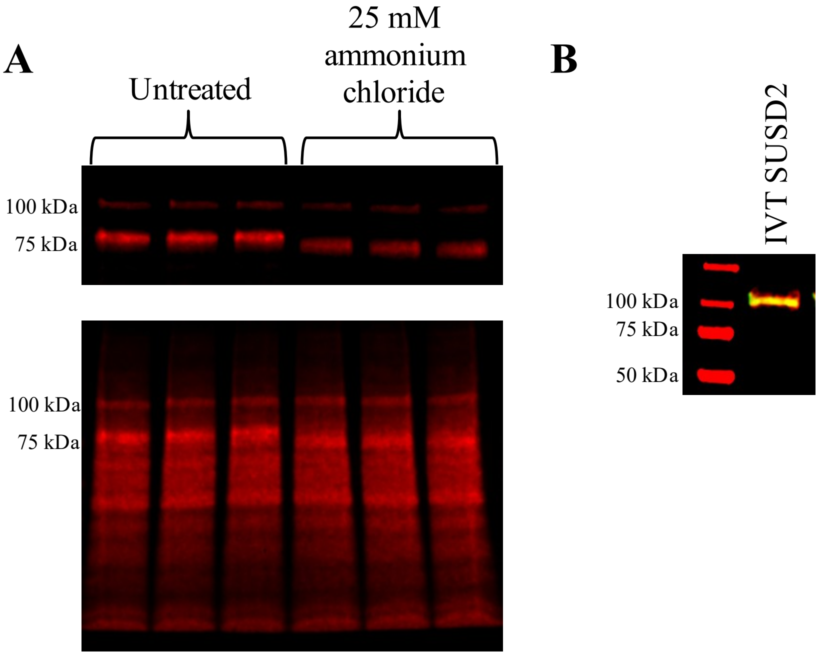

Figure S4

Figure S4. pH neutralization does not inhibit SUSD2 cleavage. (**A**) Fluorescence imaging of pulse-chase analysis (top) and REVERT total protein stain (bottom) of MDA-MB-231-SUSD2-SNAP cell lysates after neutralization of the secretory pathway using ammonium chloride. Existing SUSD2-SNAP was blocked with bromothenylpteridine (BTP), and cells were incubated for 6 h in the presence of 25 mM ammonium chloride before harvest. Harvested lysates were labeled with SNAP-surface 682 and imaged using Odyssey Fc imager. After pulse-chase imaging, the membrane was stained with REVERT total protein stain (LI-COR) and re-imaged. Samples were run in triplicate. (**B**) Western immunoblot analysis of recombinant SUSD2 produced by in vitro transcription/translation (IVT SUSD2) using anti-N- and anti-C-terminal SUSD2 antibodies. IVT SUSD2 was run on SDS-PAGE gel, transferred to PVDF membrane and labeled with SUSD2 primary antibodies. IRDye-labeled secondary antibodies were used to detect N-terminal (700 red) and C-terminal (800 green) primary antibodies.
